# Pharmacometabolomic Approach to Investigate the Response to Metformin in Patients with Type 2 Diabetes: A Cross-Sectional Study

**DOI:** 10.3390/biomedicines11082164

**Published:** 2023-08-01

**Authors:** Khaled Naja, Najeha Anwardeen, Moustafa Al-Hariri, Asmaa A. Al Thani, Mohamed A. Elrayess

**Affiliations:** 1 Biomedical Research Center, Qatar University, Doha P.O. Box 2713, Qatar; khaled.naja@qu.edu.qa (K.N.); n.anwardeen@qu.edu.qa (N.A.); aaja@qu.edu.qa (A.A.A.T.); 2 QU Health, Qatar University, Doha P.O. Box 2713, Qatar; m.al-hariri@qu.edu.qa

**Keywords:** metformin, type 2 diabetes, response variability, metabolic signatures, personalized medicine

## Abstract

Metformin constitutes the foundation therapy in type 2 diabetes (T2D). Despite its multiple beneficial effects and widespread use, there is considerable inter-individual variability in response to metformin. Our objective is to identify metabolic signatures associated with poor and good responses to metformin, which may improve our ability to predict outcomes for metformin treatment. In this cross-sectional study, clinical and metabolic data for 119 patients with type 2 diabetes taking metformin were collected from the Qatar Biobank. Patients were empirically dichotomized according to their HbA_1C_ levels into good and poor responders. Differences in the level of metabolites between these two groups were compared using orthogonal partial least square discriminate analysis (OPLS-DA) and linear models. Good responders showed increased levels of sphingomyelins, acylcholines, and glutathione metabolites. On the other hand, poor responders showed increased levels of metabolites resulting from glucose metabolism and gut microbiota metabolites. The results of this study have the potential to increase our knowledge of patient response variability to metformin and carry significant implications for enabling personalized medicine.

## 1. Introduction

Metformin constitutes the foundation therapy in type 2 diabetes due to its multiple positive effects; it is still the most commonly prescribed oral anti-diabetic medication worldwide [1]. In addition to its anti-diabetic effects, metformin is considered a potential drug for bone diseases, malignancies, neurodegenerative diseases, and recently COVID-19 [2]. Metformin has a long-term safety and efficacy profile, low risk of hypoglycemia, additive or synergistic effects in combination therapy, low cost, and wide availability [3]. Although metformin has been in use for several decades, its underlying mechanism of action, as well as its effects on metabolism, are not very well understood. Proposed mechanisms of metformin action include suppression of hepatic gluconeogenesis through activation of AMP-activated protein kinase and inhibition of mitochondrial respiration by acting on complex I [4,5]. Recent studies demonstrated that metformin alteration of the composition of the gut microbiota mediates some of its anti-diabetic effects [6,7]. The interplay between metformin and gut microbiota includes maintaining the integrity of the intestinal barrier, promoting the production of short-chain fatty acids, and regulating bile acid metabolism [8].

Despite all the positive features of metformin, the response to this drug varies significantly across individuals. Previous studies have demonstrated that metformin did not achieve optimal glycemic control in 35% of patients and that the failure rate of metformin therapy could reach 50% in newly diagnosed T2D adolescents [9,10]. Pharmacometabolomics is a rapidly developing field within metabolomics that focuses on identifying novel metabolic biomarkers associated with drug effects. It aims to provide valuable insights into the underlying mechanisms involved in drug responses and enable individualized assessment of drug therapy [11,12,13]. Gaining a comprehensive understanding of the intricate mechanisms of action of metformin holds the potential to uncover validated metabolic biomarkers. These biomarkers, in conjunction with genetic data, could facilitate the classification of individuals based on their response to the drug. This, in turn, will pave the way for personalized metformin therapy strategies. 

Our primary goal is to identify distinct metabolic signatures that are correlated with both poor and good responses to metformin. By achieving this objective, we aim to enhance our ability to predict the outcome of metformin response and potentially implement changes in patient management. To achieve this, we are conducting a retrospective cross-sectional study that focuses on the identification of novel blood metabolites associated with a response to metformin. 

## 2. Materials and Methods

### 2.1. Data Source and Study Participants

**Figure 1:**
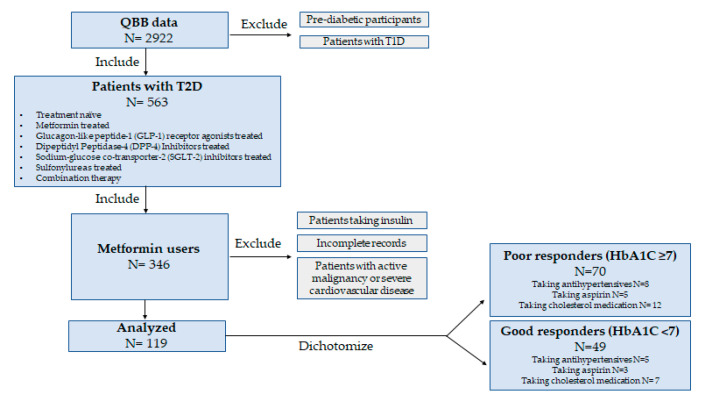
Study design.

This study obtained data from participants through the Qatar Biobank (QBB). The QBB database contains a deep phenotype of a population of Qatari nationals or long-term residents (≥15 years living in Qatar) aged 18 years and older in the State of Qatar. Extensive baseline socio-demographic data, clinical and behavioral phenotypic data, and others, including body mass index, blood pressure, glycosylated hemoglobin (HbA1c), fasting glucose level, insulin levels, lipid profile (total cholesterol, LDL, HDL, triglycerides), liver and kidney enzymes, creatinine, citric acid, lactate, and multiple other clinical biochemistry parameters were measured at the central laboratory of Hamad Medical Corporation (HMC), accredited by the College of American Pathologists. QBB data also included questionnaires related to their history of diabetes, medication usage, and metabolomics data for 1000 metabolites. This research study was approved by the Institutional Review Boards of the Qatar Biobank (QF-QBB-RES-ACC-00125). All participants provided informed consent. Among the participants, a total of 119 patients with Type 2 Diabetes who were taking metformin (daily doses range from 1000 to 2000 mg) and had available metabolic data were selected (Figure 1). Patients were empirically dichotomized according to their HbA_1C_ levels, which is the most widely used measure of glycemic control [14], into poor responders (HbA_1C_ ≥ 7) and good responders (HbA_1C_ < 7) in accordance with the American Diabetes Association guidelines and previous studies [15,16].

### 2.2. Metabolomics

All participant serum samples were subjected to untargeted metabolomics using established protocols [17]. Metabolites measurement was performed using a Thermo Scientific Q-Exactive high resolution/accurate mass spectrometer (Thermo Fisher Scientific, Inc., Waltham, MA, USA) interfaced with a heated electrospray ionization (HESI-II) source and Orbitrap mass analyzer operated at 35,000 mass resolution along with Waters ACQUITY ultra-performance liquid chromatography (UPLC) (Waters Corporation, Milford, MA, USA). A thorough explanation of the process has already been provided [17]. Hits were matched to pre-existing library entries containing over 3300 pure standard chemicals to identify compounds. Compounds were divided into several groups according to their sources. Internal standards and quality checks have been previously published [18]. In short, to adjust for discrepancies in sample preparation and instrument performance, a combination of stable isotope-labeled chemicals was utilized as internal standards. The stability and repeatability of the procedure were tracked over time using quality control samples. To reduce variability and guarantee the integrity of the samples, a systematic methodology was employed for pre-analytical sample management, including sample collection, storage, and preparation.

### 2.3. Statistical Analysis

Metabolomics data were inverse rank normalized. The software SIMCA^®^ (Version 18.0.0) [19] was used. SIMCA^®^ is a versatile multivariate data analysis software that employs advanced algorithms and interactive visualizations to explore, analyze, and interpret complex datasets. Multivariate analyses were run, including unsupervised (principal component analysis) PCA and supervised (orthogonal partial least square-discriminant analysis) OPLS-DA. R software (version 4.2.1) [20] was used to perform linear models for each metabolite (as the response variable) versus ‘poor responders’ vs. ‘good responders’ (as the explanatory variables). The model also included the following confounders: age, gender, BMI, and principal components 1 and 2. The nominal *p*-values were adjusted using the multiple testing correction method (False Discovery Rate, FDR). Statistical significance was defined as FDR < 0.05. Functional enrichment analysis was run on all *p*-value-ordered metabolite lists from the linear model performed in this study. This analysis was conducted based on a one-way Wilcoxon rank sum test followed by the FDR multiple testing correction method. These subpathways were previously defined by Metabolon through the utilization of Creative Proteomics’ technology, sophisticated bioinformatics tools, and databases to map identified metabolites onto metabolic pathways. Subpathways with less than three top hits were dropped.

## 3. Results

### 3.1. General Characteristics of Participants

One hundred and nineteen patients with T2D (55.0 ± 8.2 years) were dichotomized into ‘poor responders’ (*n* = 70) and ‘good responders’ (*n* = 49) based on their HbA_1C_ levels. Table 1 reveals significantly higher levels of fasting blood glucose, insulin, homeostatic model assessment of insulin resistance (HOMA-IR), gamma-glutamyl transferase (GGT), and triglycerides in the poor response group when compared to the good response one. 

### 3.2. Multivariate Analysis of Metabolites Differentiating Poor and Good Metformin Responders

**Figure 2:**
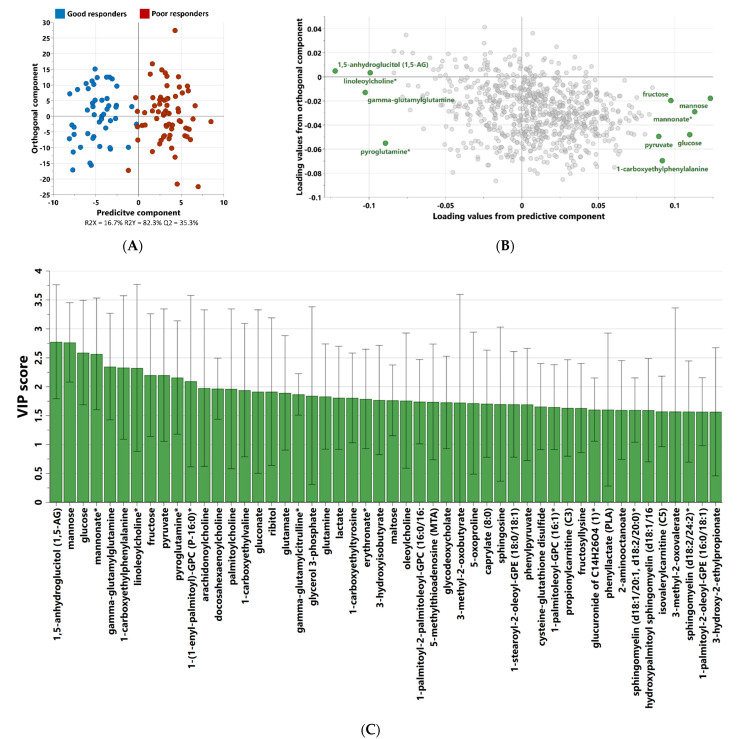
OPLS-DA score (**A**) and loading (**B**) plots depicting the most discrepant metabolites between good and poor responders among 119 patients with T2D. VIP score of the top 50 metabolites from OPLS-DA (**C**) that best differentiate components between poor and good metformin responders. (*) indicates a compound that has not been officially confirmed based on a standard but that Metabolon is confident in its identity.

Non-targeted metabolomics analysis was performed to investigate the metabolic signatures of 119 patients with Type 2 Diabetes (T2D) taking metformin. OPLS-DA was used to identify the best distinguishing components between poor and good responders, as shown in Figure 2. OPLS-DA showed one predictive and two orthogonal components, with the discriminatory component accounting for 82.3% of the variance between poor and good responders. Figure 2C shows the list of metabolites with VIP > 1.5.

### 3.3. Univariate Analysis of Metabolites Differentiating Poor and Good Metformin Responders

Linear model analysis revealed a number of FDR (≤0.05) significant changes between the two studied groups (Table 2). This includes 1,5-anhydroglucitol, glucose, mannose, and pyruvate. Changes were also seen in microbiota-related metabolites, including 1-carboxyethylphenylalanine, mannonate, and methyl glucopyranoside (α+β). Other metabolites differentiating the two groups also included glutamine, gamma-glutamylglutamine, glutamate, and pyroglutamine. In addition, many sphingomyelins and acylcholines were also found to differentiate between the two groups.

### 3.4. Functional Enrichment Analysis

**Figure 3:**
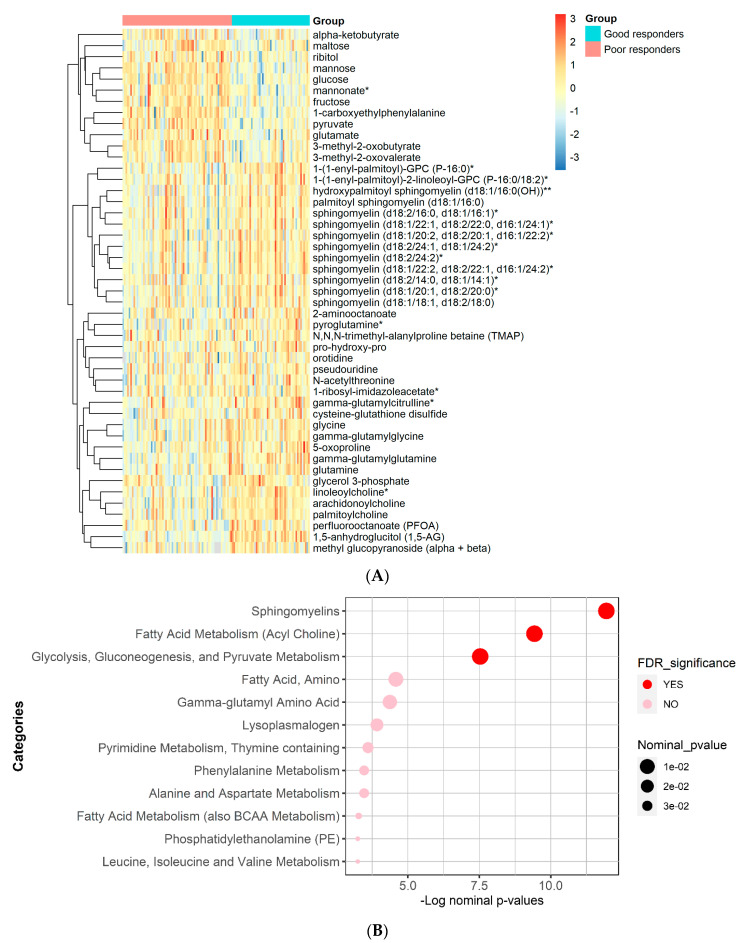
Heatmap and functional enrichment analysis from comparing metformin good vs. poor responders. (**A**) Heatmap representing the metabolites with significantly different levels (FDR ≤ 0.05). (**B**) Results from functional enrichment analysis based on metabolite ranks by *p*-value using the Wilcoxon rank sum test. (*) indicates a compound that has not been officially confirmed based on a standard but that Metabolon is confident in its identity.

The results of functional enrichment analyses (Figure 3) indicated significant differences in sphingomyelins, fatty acid metabolism (Acyl Choline), glycolysis, gluconeogenesis, and pyruvate metabolism. A heatmap showing the top metabolites is shown in Figure 3.

## 4. Discussion

Metformin is used on a daily basis by more than 200 million patients with T2D worldwide. Despite its multiple beneficial effects and widespread use, there is considerable inter-individual variability in response to metformin. Genetic variation may be one of the important determinants explaining the variation in individual responses to metformin. However, it is now estimated that genetic background accounts for only 20–40% of the inter-individual variability in response to drugs [12]. Pharmacometabolomics is a powerful tool to explain the differences in drug response among individuals since it is sensitive to both genetic and environmental factors such as diet and the patient’s microbiome. In this retrospective study, we identified metabolic signatures associated with poor and good responses to metformin in a set of 119 samples from the Qatar Biobank. These interpretations of these differences and the potential impact on metformin response are summarized below.

### 4.1. Glycolysis, Gluconeogenesis, and Pyruvate Metabolism

Univariate and pathway enrichment analyses showed, expectedly, that the blood sugars and metabolites related to glycolysis, gluconeogenesis, and pyruvate metabolism (glucose, fructose, mannose, and pyruvate) were significantly higher in the poor response group, reflecting a greater level of hyperglycemia, the impaired action of metformin to inhibit gluconeogenesis, and dysregulated glucose metabolism. This is consistent with the previous literature [21], in particular, the recent QBB findings [22]. Our emerging results showed that 1,5-anhydroglucitol (1,5-AG) was significantly higher in good responders. Gormsen et al. [23] reported that 1,5-AG was associated with the glucose-lowering effect of metformin. Similarly, Villena Chávez et al. recently reported, in a study involving one hundred outpatients with T2D, that patients with HbA1c < 7% had significantly higher 1,5-AG than those with HbA1c ≥ 7% [24].

### 4.2. Gut Microbiome Metabolites

Our emerging data showed that gut microbiome-derived metabolites, namely, mannonate, 1-carboxyethylphenylalanine, and methyl glucopyranoside (α+β), were associated with metformin response. Additionally, more gut metabolites were associated with the response to metformin, including 1-carboxyethyltyrosine, 1-carboxyethylvaline, phenylacetate, and phenyllactate, although they did not reach the FDR level of significance. Indeed, the gut microbiome is a vital component that needs to be given more attention since it plays a substantial role in drug response and effectiveness by altering the activity, toxicity, and bioavailability of therapeutic drugs [25,26]. The crosstalk between gut microbiota metabolism and metformin is now well established, and it is now clear that the gut microbiota participates in the glucose-lowering effects of metformin. In a clinical study involving healthy individuals, the hypoglycemic effect of metformin was correlated with the microbiome through specific changes in metabolites [27]. These metabolites can derive directly from bacteria or the transformation of dietary or host-derived substrates [28]. Examples include 1-carboxyethyltyrosine, 1-carboxyethylvaline, and 1-carboxyethylphenylalanine, which are organic compounds that belong to the class of amino acid derivatives. Methyl glucopyranoside is a monosaccharide derived from glucose that can exist in two forms: alpha and beta.

An increase in the production of short-chain fatty acids (SCFAs), regulation of bile acid metabolism, and improvement of glucose homeostasis are among the proposed mechanisms by which metformin exerts part of its hypoglycemic effects through the gut microbiota [8]. However, the relationship between metformin and the microbiome is bidirectional, and the metabolites produced by the gut microbiota could also influence the efficacy of metformin and contribute to the inter-individual difference in response to the drug [29]. The gut microbiota can produce hundreds of metabolites [30], yet studies addressing the influence of these metabolites on metformin response remain very scarce. Koh et al. showed that imidazole propionate, a microbial metabolite, is higher in T2D patients treated with metformin [31]; the same study also showed that imidazole propionate impairs the glucose-lowering effect of metformin in mice. On the other hand, Sun et al. [32] showed that the bile acid glycoursodeoxycholic acid (GUDCA) mediates the glucose-lowering effect of metformin by binding to the nuclear receptors FXR. 

Shannon diversity is one of the most common alpha diversity metrics reported in the gut microbiome literature. It summarizes taxonomic richness and evenness, and it has been suggested as a marker for microbiome health [33]. Wilmanski et al. [34] classified 1-carboxyethylphenylalanine and methyl glucopyranoside (α+β) among the 11 strongest predictors of the gut microbiome Shannon α-diversity. Interestingly, 1-carboxyethylphenylalanine associated in our study with the poor responder group was identified by Wilmanski to be associated with less microbiome diversity, while methyl glucopyranoside (α+β) associated in our study with the good responders, was identified to be associated with more microbiome diversity and a healthier status. Additionally, our previous studies on QBB data from non-diabetic individuals showed that 1-carboxyethylphenylalanine was identified as the most discriminating metabolite of insulin resistance [35,36]. Our results suggest that dysbiosis in gut microbiota is associated with a reduced response to metformin, and improving gut health could improve the effectiveness of metformin. Relatedly, Şahin et al. showed that patients treated with metformin combined with probiotics had a greater reduction in HbA_1C_ from baseline compared to patients treated with metformin only [37]. However, the extent to which a certain microbiome profile is necessary for the metformin impact remains unknown, and more validation studies are needed. 

Our results showed that mannonate, an *E. coli* K-12 metabolite [38], was associated with poor response to metformin. Previous data on mannonate is very scarce. *E. coli* K-12 is considered an opportunistic commensal gut microbe and was recently discovered to distort the barrier integrity in human intestinal cells [39]. Knowing that metformin exerts part of its hypoglycemic effects by altering the gut microbiota in ways that maintain the integrity of the intestinal barrier [40], mannonate may exert an opposite role of metformin action by hindering the function of the intestinal barrier. The diet and potential metabolites produced during different types of diet may play a role in this context [41]. Further studies are needed to study the effect of metformin on intestinal barriers in relation to Shannon diversity.

### 4.3. Sphingomyelins

Our emerging results showed an increase in all sphingomyelins in the responsive group, and the enrichment analysis showed an FDR-significant pathway of sphingomyelins. Sphingomyelin is one of the main phospholipids that make up the cell plasma membranes, where it forms—with cholesterol—lipid rafts. The latter serve as platforms for protein assemblies involved in signal transduction. Additionally, sphingomyelin is the most abundant sphingolipid in plasma lipoproteins, and it plays a role as an active center for lipid and glucose metabolism [42,43]. Aligned with our data, a study that compared metabolic signatures associated with T2D and impaired fasting glucose showed that sphingomyelins were significantly reduced in the disease group [44]. Additionally, data from the QBB analyzed by Zaghlool et al. [22] and Yousri et al. [45] reported low levels of two sphingomyelins in T2D and severe insulin-deficient diabetes. Two other studies analyzed the serum metabolite profile associated with the incidence of T2D using a targeted metabolomic approach and identified many sphingomyelins related to a low incidence of T2D [46,47]. Moreover, a study showed that downregulated metabolism of sphingomyelins can affect insulin sensitivity and lead to β cell dysfunction [48]. A study on sphingomyelin synthase 1-null mice showed that reduced sphingomyelin synthesis is associated with increased reactive oxygen species and reduced insulin secretion [49]; another study compared the lipidomic profiles in monkeys with and without diabetes and showed reduced sphingomyelins in the presence of biochemical profiles suggestive of reduced insulin sensitivity [50]. Moreover, Sharma et al. [51] reported low levels of ceramides associated with a beneficial metabolic response to metformin. Of note, a study testing the role of metformin in ovarian cancer suggested that the sphingolipid rheostat may be a novel metabolic target of metformin [52], and metformin alleviated inflammation by targeting sphingolipid metabolism through inhibiting sphingosine kinase 1 (SPHK1), an enzyme which converts sphingosine, a product of sphingomyelin, to sphingosine 1-phostpate [52,53]; the latter has been shown to act via stimulation of the sphingosine-1-phosphate receptor-2 to impair insulin signaling and reduce hepatic insulin resistance [54]. However, our results did not show any significant difference in sphingosine 1-phosphate levels between the two groups. A recent review article showed that the role of SPHK1 in insulin resistance is controversial [55]. Although our results highlight the potential use of sphingomyelins to assess the response to metformin, a more precise and comprehensive measurement of sphingomyelin composition in different metformin treatment contexts should be performed.

### 4.4. Glutamine Metabolism

In this study, glutamate and glutamine were associated with poor and good responses to metformin, receptively, consistent with a Japanese study that showed a positive correlation between homeostasis model assessment of insulin resistance (HOMA-IR) and glutamate but a negative correlation between glutamine and glycine [56]. The findings were also consistent with another study that suggested glutamate to be among the baseline metabolites associated with HOMA-IR [57]. Moreover, Greenfield et al. showed that glutamine supplementation was associated with improved glucose tolerance [58], Cheng et al. showed that a high glutamine/glutamate ratio was associated with a lower risk of T2D incidence [59], whereas Liu et al. showed that lower levels of glutamine and higher levels of glutamate were associated with increased risk of T2D [60]. However, d’Almeida et al. showed that glutamate was lower in patients with worse glycemic control [61]. Interestingly, many studies demonstrated that metformin modifies glutamine metabolism and reduces glutamate accumulation by inhibiting glutaminase, the enzyme which converts glutamine to glutamate [62,63]. Noteworthy, glutaminase is overexpressed in cancer cells [64], and this explains why metformin is beneficial for some patients with cancer. Many studies target glutamine metabolism as a potential therapeutic strategy for cancer [65]. Though further validation is required, the glutamine-to-glutamate ratio could be clinically important as a potential metabolic marker of a patient’s sensitivity to metformin.

### 4.5. Choline Metabolism

Our data showed an association between linoleoylcholine and arachidonoylcholine with good response to metformin, and enrichment analysis showed an FDR significant association of metformin response to the acylcholine pathway. Indeed, normal human plasma contains, among choline derivatives, those that are acylated with unsaturated fatty acid residues (e.g., arachidonic and linoleic), which are a recently discovered family of endogenous lipids [66]. Little is known about the biological activity of acylcholines, but a recent study demonstrated that arachidonoylcholine inhibits the human erythrocyte acetylcholinesterase and could act as an endogenous modulator of the acetylcholine signaling system. [67]. A study showed that metformin was found to moderately inhibit the activity of acetylcholinesterase [68]. Markowicz-Piasecka et al. [69] concluded that metformin has an influence on the cholinergic system in the brain and may play an important role in the treatment of neurodegenerative diseases. This could also explain metformin′s appetite-suppressing effects through cholinergic pathways identified in the brain [70].

### 4.6. Other Metabolites

Five metabolites involved in the glutathione metabolism (gamma-glutamylglutamine, gamma-glutamylglycine, cysteine-glutathione disulfide, pyroglutamine, and 5-oxoproline) were higher in the good responder group indicating adequate glutathione metabolism and emphasizing anti-oxidative properties of metformin. 

This also could justify the high levels of glutamate in the poor response group. In fact, glutamate is metabolized to glutathione via two steps [71]. Variants in the genes coding for the enzyme glutamate cysteine ligase, which catalyzes the first step of glutathione synthesis, have been shown to confer protection against T2D through increased secretion of glutathione [72]. However, glutathione was not among the measured metabolites in this study, and further studies are needed to elucidate the possible relationship between glutathione and metformin response.

Our data showed that only 41% of patients achieved acceptable glycemic control upon metformin treatment. Many studies reported a low percentage of patients who achieved glycemic control, and poor response to metformin could be one of the possible reasons [73,74]. Our clinical data showed that HOMA-IR was significantly higher in the poor responder group. This is confirmed by the many metabolites discussed above that are related to insulin resistance. Results from Qatari patients with severe insulin resistance showed that these patients did not respond adequately to insulin sensitizers such as metformin [75]. This may explain the low efficacy of metformin in the case of severe insulin resistance. It also highlights the importance of clustering T2D patients, before treatment initiation, according to the sub-stratification of Ahlqvist et al. [76], who used six variables to stratify patients into four clusters representing T2D subtypes.

## 5. Conclusions

This study provides the research community with a wealth of novel metabolic signatures associated with response to metformin, which has the potential to identify pathways involved in the action of the drug and predict therapeutic outcomes. One limitation of this study is the cross-sectional nature of the cohort of metformin-treated subjects with T2D. It is unclear whether those with higher HbA1C values actually responded poorly to metformin or had more severe diabetes before treatment with the drug; accordingly, some metabolites could be associated with the stage or complications of diabetes rather than a poor or good response to metformin. Moreover, the treatment time with metformin was not available. A longitudinal cohort study will thus be required to validate our identified biomarkers. Another limitation is the small sample size; confirming our findings in a larger cohort is thus warranted. We believe these results can be translated into clinically applicable metabolic biomarkers, which may enable further studies investigating personalized therapy approaches.

## Figures and Tables

**Table 1:** General Characteristics of Participants.

Test	Variable	Total (*N* = 119)	Poor Responders (*N* = 70)	Good Responders (*N* = 49)	*p* Value
Vital signs	Gender (M/F)	58/61	34/36	24/25	0.56
	Age	55.0 (8.2)	55.3 (8.2)	54.6 (8.3)	0.67
	BMI (kg/m^2^)	31.2 (27.2–35.2)	31.0 (26.8–36.7)	31.5 (27.9–33.9)	0.93
	Systolic blood pressure (mmHg)	126.6 (14.4)	127.9 (14.7)	124.9 (14.1)	0.27
	Diastolic blood pressure (mmHg)	74.0 (10.8)	73.1 (11.0)	75.4 (10.4)	0.25
	Pulse rate	73.2 (11.9)	76.9 (11.4)	67.9 (10.5)	<0.001
Blood sugar	Fasting blood glucose (mmol/L)	8.6 (6.6–11.2)	10.8 (9.1–15.0)	6.4 (5.7–7.8)	<0.001
	HbA_1C_ (%)	7.5 (6.7–9.0)	8.5 (8.0–9.3)	6.5 (6.1–6.8)	<0.001
	Insulin (uU/mL)	15.0 (10–26.4)	18.7 (12.0–35.6)	12.0 (8.7–16.5)	<0.001
	HOMA-IR	6.5 (3.3–12.9)	8.6 (5.7–17.2)	3.3 (2.4–5.80)	<0.001
	C-peptide (ng/mL)	3.0 (2.0–3.9)	3.2 (2.0–4.0)	2.5 (2.0–3.7)	0.21
Lipid profile	Total cholesterol (mmol/L)	4.50 (3.9–5.0)	4.63 (3.80–5.30)	4.40 (3.89–4.98)	0.45
	HDL-cholesterol (mmol/L)	1.12 (1.00–1.33)	1.12 (0.98–1.30)	1.14 (1.03–1.34)	0.34
	LDL-cholesterol (mmol/L)	2.56 (2.00–3.00)	2.56 (2.00–3.20)	2.65 (2.00–3.00)	0.85
	Triglyceride (mmol/L)	1.60 (1.20–2.20)	1.60 (1.36–2.36)	1.24 (1.09–1.80)	0.002
Kidney function	Creatinine (µmol/L)	66.0 (55.0–77.0)	67.0 (53.0–77.0)	65.0 (54.0–79.5)	0.85
	Urea (mmol/L)	4.5 (3.9–5.5)	4.6 (4.0–5.6)	4.4 (3.6–5.1)	0.12
	Lactate (mmol/L)	0.9 (0.7–1.3)	0.9 (0.7–1.2)	0.9 (0.7–1.3)	0.84
	Bicarbonate (mmol/L)	26.6 (2.3)	26.2 (2.2)	27.1 (2.4)	0.029
	Total protein (g/L)	72.3 (3.5)	72.8 (3.7)	71.6 (3.1)	0.056
	Uric acid (µmol/L)	285 (239–336)	282 (239–309)	296 (238–392)	0.084
Liver function	Albumin (g/L)	44 (43–46)	44 (43–46)	45 (42–45)	0.78
	ALT (U/L)	20 (15–30)	21 (16–30)	19 (14–29.5)	0.19
	AST (U/L)	17 (14–21)	17 (14–22)	16 (13.5–21)	0.37
	GGT (U/L)	23 (15–33)	29 (21–34)	17 (12–27)	0.023
Hormones	TSH (mIU/L)	1.43 (0.97–2.13)	1.52 (0.95–2.20)	1.41 (1.02–2.02)	0.80
	Free thyroxine (pmol/L)	13.2 (12.3–14.2)	13.2 (12.2–14.1)	12.9 (12.3–14.4)	0.90
	Free triiodothyronine (pmol/L)	4.22 (0.59)	4.22 (0.56)	4.21 (0.63)	0.89

Data are presented as mean (SD), median (IQR), and number for parametric, non-parametric, and nominal variables, respectively. The difference between the mean/median was evaluated using an independent *t*-test/Mann–Whitney U test as appropriate. The chi-square test was used for the nominal variable. Abbreviations: BMI, body mass index; HbA_1C_, glycated hemoglobin; HOMA-IR, homeostatic model assessment of insulin resistance; HDL, high-density lipoprotein; LDL, low-density lipoprotein; ALT, alanine transaminase; AST, aspartate aminotransferase; GGT, gamma-glutamyl transferase; TSH, Thyroid stimulating hormone.

**Table 2:** Metabolites differentiating metformin from good vs. poor responders, correcting for age, gender, BMI, and principal components 1 and 2.

Metabolite	Super-Pathway	Subpathway	Estimate	SE	*p*-Value	FDR
1,5-anhydroglucitol (1,5-AG)	Carbohydrate	Glycolysis, Gluconeogenesis, and Pyruvate Metabolism	1.164	0.160	5.89 × 10^−11^	5.16 × 10^−8^
Mannose	Carbohydrate	Fructose, Mannose, and Galactose Metabolism	−0.942	0.159	3.73 × 10^−8^	1.63 × 10^−5^
Pyroglutamine *	Amino Acid	Glutamate Metabolism	0.898	0.154	6.92 × 10^−8^	2.02 × 10^−5^
Glucose	Carbohydrate	Glycolysis, Gluconeogenesis, and Pyruvate Metabolism	−0.758	0.145	8.87 × 10^−7^	0.000194
Linoleoylcholine *	Lipid	Fatty Acid Metabolism (Acyl Choline)	0.862	0.169	1.42 × 10^−6^	0.00025
Gamma-glutamylglutamine	Peptide	Gamma-glutamyl Amino Acid	0.871	0.183	5.67 × 10^−6^	0.000827
1-carboxyethylphenylalanine	Amino Acid	Phenylalanine Metabolism	−0.579	0.123	6.99 × 10^−6^	0.000875
Mannonate *	Xenobiotics	Food Component/Plant	−0.660	0.142	9.41 × 10^−6^	0.00103
Sphingomyelin (d18:2/24:2) *	Lipid	Sphingomyelins	0.699	0.157	2.08 × 10^−5^	0.002027
1-(1-enyl-palmitoyl)-GPC (P-16:0) *	Lipid	Lysoplasmalogen	0.748	0.173	3.38 × 10^−5^	0.002963
Hydroxypalmitoyl sphingomyelin (d18:1/16:0(OH)) **	Lipid	Sphingomyelins	0.670	0.161	6.52 × 10^−5^	0.004444
Arachidonoylcholine	Lipid	Fatty Acid Metabolism (Acyl Choline)	0.734	0.177	6.85 × 10^−5^	0.004444
Pyruvate	Carbohydrate	Glycolysis, Gluconeogenesis, and Pyruvate Metabolism	−0.704	0.171	7.10 × 10^−5^	0.004444
3-methyl-2-oxobutyrate	Amino Acid	Leucine, Isoleucine and Valine Metabolism	−0.743	0.181	7.90 × 10^−5^	0.004612
Palmitoyl sphingomyelin (d18:1/16:0)	Lipid	Sphingomyelins	0.543	0.136	0.00012	0.006159
Sphingomyelin (d18:2/24:1, d18:1/24:2) *	Lipid	Sphingomyelins	0.559	0.140	0.000124	0.006159
Pseudouridine	Nucleotide	Pyrimidine Metabolism, Uracil containing	0.689	0.174	0.000128	0.006159
Glutamate	Amino Acid	Glutamate Metabolism	−0.574	0.145	0.000134	0.006159
Sphingomyelin (d18:1/20:1, d18:2/20:0) *	Lipid	Sphingomyelins	0.592	0.151	0.000153	0.006721
Sphingomyelin (d18:1/22:2, d18:2/22:1, d16:1/24:2) *	Lipid	Sphingomyelins	0.551	0.143	0.000189	0.007892
2-aminooctanoate	Lipid	Fatty Acid, Amino	0.631	0.164	0.0002	0.007967
Alpha-ketobutyrate	Amino Acid	Methionine, Cysteine, SAM and Taurine Metabolism	−0.709	0.184	0.000209	0.007967
Palmitoylcholine	Lipid	Fatty Acid Metabolism (Acyl Choline)	0.661	0.174	0.000246	0.008988
Glutamine	Amino Acid	Glutamate Metabolism	0.673	0.181	0.000326	0.011422
Gamma-glutamylcitrulline *	Peptide	Gamma-glutamyl Amino Acid	0.679	0.184	0.000347	0.011683
N-acetylthreonine	Amino Acid	Glycine, Serine, and Threonine Metabolism	0.572	0.155	0.000361	0.011699
Fructose	Carbohydrate	Fructose, Mannose, and Galactose Metabolism	−0.577	0.157	0.000384	0.01202
Cysteine-glutathione disulfide	Amino Acid	Glutathione Metabolism	0.584	0.161	0.000434	0.013119
Pro-hydroxy-pro	Amino Acid	Urea cycle; Arginine and Proline Metabolism	0.640	0.185	0.000774	0.021566
Sphingomyelin (d18:1/20:2, d18:2/20:1, d16:1/22:2) *	Lipid	Sphingomyelins	0.495	0.143	0.000776	0.021566
N, N, N-trimethyl-alanylproline betaine (TMAP)	Amino Acid	Urea cycle; Arginine and Proline Metabolism	0.516	0.149	0.000788	0.021566
Methyl glucopyranoside (alpha + beta)	Xenobiotics	Food Component/Plant	0.721	0.213	0.001034	0.026924
Glycerol 3-phosphate	Lipid	Glycerolipid Metabolism	0.670	0.199	0.001045	0.026924
Perfluorooctanoate (PFOA)	Xenobiotics	Chemical	0.555	0.166	0.001141	0.028559
Glycine	Amino Acid	Glycine, Serine and Threonine Metabolism	0.661	0.200	0.001269	0.030868
1-(1-enyl-palmitoyl)-2-linoleoyl-GPC (P-16:0/18:2) *	Lipid	Plasmalogen	0.470	0.144	0.001466	0.034713
Sphingomyelin (d18:2/14:0, d18:1/14:1) *	Lipid	Sphingomyelins	0.377	0.117	0.001664	0.038324
Sphingomyelin (d18:2/16:0, d18:1/16:1) *	Lipid	Sphingomyelins	0.377	0.117	0.001719	0.038324
3-methyl-2-oxovalerate	Amino Acid	Leucine, Isoleucine and Valine Metabolism	−0.582	0.181	0.00175	0.038324
5-oxoproline	Amino Acid	Glutathione Metabolism	0.575	0.180	0.001873	0.039081
Maltose	Carbohydrate	Glycogen Metabolism	−0.483	0.152	0.001874	0.039081
Orotidine	Nucleotide	Pyrimidine Metabolism, Orotate containing	0.522	0.165	0.002111	0.042617
1-ribosyl-imidazoleacetate *	Amino Acid	Histidine Metabolism	0.438	0.139	0.002141	0.042617
Gamma-glutamylglycine	Peptide	Gamma-glutamyl Amino Acid	0.625	0.200	0.002242	0.043638
Sphingomyelin (d18:1/22:1, d18:2/22:0, d16:1/24:1) *	Lipid	Sphingomyelins	0.390	0.126	0.002427	0.046226
Sphingomyelin (d18:1/18:1, d18:2/18:0)	Lipid	Sphingomyelins	0.462	0.150	0.002696	0.049799
Ribitol	Carbohydrate	Pentose Metabolism	−0.516	0.168	0.002729	0.049799

Estimate represents beta value. Abbreviations: SE, standard error; FDR, false discovery rate. (*) indicates a compound that has not been officially confirmed based on a standard but that Metabolon is confident in its identity.

## Data Availability

The datasets used and/or analyzed during the current study are available from the corresponding author upon reasonable request.
